# Evolutionary selected Tibetan variants of HIF pathway and risk of lung cancer

**DOI:** 10.18632/oncotarget.14340

**Published:** 2016-12-28

**Authors:** Lucie Lanikova, N. Scott Reading, Hao Hu, Tsewang Tashi, Tatiana Burjanivova, Anna Shestakova, Bhola Siwakoti, Binay Kumar Thakur, Chin Bahadur Pun, Amir Sapkota, Sarah Abdelaziz, Bing-Jian Feng, Chad D. Huff, Mia Hashibe, Josef T. Prchal

**Affiliations:** ^1^ Division of Hematology, University of Utah School of Medicine, Salt Lake City, Utah, USA; ^2^ Institute of Molecular Genetics, Academy of Sciences, Prague, Czech Republic; ^3^ Institute for Clinical and Experimental Pathology, ARUP Laboratories, Salt Lake City, Utah, USA; ^4^ Department of Pathology, University of Utah School of Medicine, Salt Lake City, Utah, USA; ^5^ University of Texas, MD Anderson Cancer Center, Houston, Texas, USA; ^6^ Department of Molecular Biology, Comenius University in Bratislava, Jessenius Faculty of Medicine in Martin, Slovak Republic; ^7^ B.P. Koirala Memorial Cancer Hospital, Bharatpur, Chitwan, Nepal; ^8^ Maryland Institute for Applied Environmental Health, and University of Maryland College Park School of Public Health, Maryland, USA; ^9^ Division of Public Health, Department of Family & Preventive Medicine and Huntsman Cancer Institute, University of Utah School of Medicine, Salt Lake City, Utah, USA; ^10^ Department of Dermatology, University of Utah School of Medicine, Salt Lake City, Utah, USA; ^11^ Department of Pathophysiology and 1st Department of Medicine, 1st Faculty of Medicine, Charles University in Prague, Czech Republic

**Keywords:** hypoxia, ENGL1/PHD2, EPAS1/HIF-2α, high-altitude adaptation, lung cancer

## Abstract

Tibetans existed in high altitude for ~25 thousand years and have evolutionary selected unique haplotypes assumed to be beneficial to hypoxic adaptation. *EGLN1*/PHD2 and *EPAS1*/HIF-2α, both crucial components of hypoxia sensing, are the two best-established loci contributing to high altitude adaptation. The co-adapted Tibetan-specific haplotype encoding for PHD2:p.[D4E/C127S] promotes increased HIF degradation under hypoxic conditions. The Tibetan-specific 200 kb *EPAS1* haplotype introgressed from an archaic human population related to Denisovans which underwent evolutionary decay; however, the functional variant(s) responsible for high-altitude adaptation at *EPAS1*/HIF-2α have not yet been identified. Since HIF modulates the behavior of cancer cells, we hypothesized that these Tibetan selected genomic variants may modify cancer risk predisposition. Here, we ascertained the frequencies of *EGLN1*^D4E/C127S^ and *EGLN1*^C127S^ variants and ten *EPAS1*/HIF-2α variants in lung cancer patients and controls in Nepal, whose population consists of people with Indo-Aryan origin and Tibetan-related Mongoloid origin. We observed a significant association between the selected Tibetan *EGLN*1/PHD2 haplotype and lung cancer (p=0.0012 for D4E, p=0.0002 for C127S), corresponding to a two-fold increase in lung cancer risk. We also observed a two-fold or greater increased risk for two of the ten *EPAS1*/HIF-2α variants, although the association was not significant after correcting for multiple comparisons (p=0.12). Although these data cannot address the role of these genetic variants on lung cancer initiation or progression, we conclude that some selected Tibetan variants are strongly associated with a modified risk of lung cancer.

## INTRODUCTION

High altitude is one of the most challenging environments for human existence. Nevertheless, long-term high-altitude populations have adapted through evolutionary mechanisms to survive for millennia in environments with extremely low atmospheric oxygen pressure (hypoxia). The molecular basis of such adaptation is still not completely understood. In Tibetans, alterations in hypoxia inducible transcription factor (HIF) pathway have been shown to be such an adaptive response [1–3]. Genome-wide scans for positive selection in Tibetan population revealed several evolutionarily selected genetic loci which may allow adaptive biological changes in humans (reviewed in [4]). Two Tibetan specific loci with the highest prevalence and strongest fixation index (F_ST_)[5] are *EGLN1* encoding prolyl hydroxylase 2 (PHD2) - a principal negative regulator of the HIFs levels, and a large genomic region including *EPAS1* which encodes HIF-2α - an isoform of one of the three α-subunits of HIFs (encoded by *EPAS1* gene); both these genomic loci were reported to protect from polycythemia [3, 6–8].

We previously identified *EGLN1/*PHD2, p.D4E (*NM_022051:c.12C>G*), a coding variant with functional impact, that is present in *cis* with the previously reported variant p.C127S (*NM_022051:c.380G>C*, rs12097901). This Tibetan-specific selected variant *EGLN1*^D4E/C127S^ has high gene frequency (~80%) among Tibetans [7, 9] and is a gain-of-function variant of PHD2 in hypoxia resulting in downregulation of HIF-1 and HIF-2. However, in another report using a different methodology, this variant was described as a loss-of-function (hypomorphic) variant leading to augmented HIF activation [10]. Several studies of high-altitude adaptation have reported that Tibetan *EPAS1/*HIF-2α haplotype was influenced by strong positive selection in Tibet [reviewed in [4]] and protection from polycythemia [3, 11]. The Tibetan advantageous *EPAS1* haplotype originated from an archaic *Homo* population related to Denisovans. The adaptive *EPAS1* haplotype entered the Tibetan population via archaic introgression and then increased to a very high frequency among Tibetans due to positive selection [12]. Because the advantageous *EPAS1* haplotype diverged from the Denisovan haplotype approximately one million years ago, there are many Tibetan-specific variants in the *EPAS1* region that are absent from the Denisovan reference genome [5, 13]. The functional variant(s) contributing to high-altitude adaptation on the *EPAS1* haplotype is (are) currently unknown.

HIFs control the expression of a large array of genes in order to mediate hypoxia response, including those that promote glucose uptake, facilitate glycolysis, stimulate angiogenesis, or regulate apoptosis and erythropoiesis [14]. HIFs isoforms have similar structure and share important, but non-redundant role in hypoxia regulation. Despite having closely related and conservative protein domains, they have distinct and tissue-specific gene expression patterns. Whereas HIF-1α is expressed ubiquitously, the expression of HIF-2α is restricted to certain tissues and is especially important for mediating erythropoiesis, vascularization, and pulmonary development [15]. The pleiotropic effect of HIFs is underlined by diseases in which they have both protective responses (i.e. coronary artery disease, peripheral artery disease, colitis) and also contribute to pathogenesis (i.e. congenital polycythemias, cancer, pulmonary arterial hypertension). In many solid tumors the increased HIF-1α and/or HIF-2α levels in diagnostic tumor biopsies are associated with poor prognosis and increased risk of mortality due to their invasive and metastatic properties [16].

Due to the conflicting data on whether HIFs are uniformly tumor initiators or suppressors [17, 18], or whether HIFs function differently depending on tissue context, we hypothesized that Tibetan selected genomic variants may modify cancer risk predisposition. We therefore genotyped patients and controls from a case-control study for lung cancer in Nepal. Nepal is a land of diverse ethnic groups, but they can be organized into two major groups: Indo-Aryan origin from the Indian subcontinent, and those belonging to the Tibetan-related Mongoloid race from the Himalayas. Since most of the Tibetan-related mongoloid Nepalese and Tibetans share the same genetic background [13], we believe they would also have the Tibetan-specific *EGLN1* and *EPAS1* genetic variants. We evaluated the association between lung cancer and Tibetan-specific *EGLN1* and *EPAS1* genetic variants. To do so, we genotyped the two *EGLN1* variants, *EGLN1*^D4E^ and *EGLN1*^C127S^ and ten *EPAS1* SNPs with strong evidence of positive selection.

## RESULTS

The study was carried out on 214 lung cancer patients (114 male, 53%; 100 female, 47%) and 213 controls (129 male, 61%; 84 female, 39%). The demographic characteristics are shown in Table 1. The distribution of self-reported race/ethnicity, religion and residence are shown since these are variables that we need to consider as potential confounders. The majority of both cases and controls are Hindu and live in the plains regions. Approximately 2.8% of cases and 1.9% of controls live in the high altitude mountain region. Both the cases and controls were widely distributed across the western to eastern regions of Nepal for their residence.

**Table 1:** Demographic characteristics of lung cancer patients and controls

	Patients		Controls	
TOTAL	214		213	
Age				
<50	28	13.1%	50	23.5%
50-59	50	23.4%	75	35.2%
60-69	93	43.5%	65	30.5%
70+	43	20.1%	23	10.8%
**Sex**				
Male	114	53.3%	129	60.6%
Female	100	46.7%	84	39.4%
**Residence**				
Plains	116	54.2%	153	71.8%
Hills	92	43.0%	56	26.3%
Mountains	6	2.8%	4	1.9%
**Race/Ethnicity**				
Brahmin	39	18.2%	65	30.5%
Chettri	41	19.2%	37	17.4%
Rai, Limbu, Magar	58	27.1%	15	7.0%
Tharu, Madishe	36	16.8%	34	16.0%
Other	40	18.7%	62	29.1%
**Religion**				
Hindu	198	92.5%	188	88.3%
Other	16	7.5%	25	11.7%
**Residence**				
Far west	8	3.7%	8	3.8%
Mid west	24	11.2%	18	8.5%
West	71	33.2%	49	23.0%
Central	35	16.4%	83	39.0%
East	28	13.1%	26	12.2%

All samples were genotyped using a high resolution melting assay and then validated by Sanger sequencing. Among 427 total samples, 20 healthy controls and 40 patients were heterozygous or homozygous for *EGLN1*^D4E^. As previously observed [9], all individuals with *EGLN1*^D4E^ were also heterozygous or homozygous for *EGLN1*^C127S^, i.e. *EGLN1*^D4E^ has always been observed in *cis* with *EGLN1*^C127*S*^. The *EGLN1*^D4E/C127S^ haplotype was associated with an approximately two-fold increase in lung cancer risk (p=0.0012, p=0.0002) (Table 2). The associations remained significant after adjusting for genotypes at the *EPAS1* locus (p=0.0094, p=0.0022) (Table 2). We further investigated the *EGLN1*^D4E/C127S^ variant by adjusting for smoking status and the associations still held (Table 3). The association between the *EGLN1*^D4E/C127S^ variants and lung cancer risk was observed among men, by study participants residing in the West of Nepal and reported to live in the high altitude, and among Hindus.

**Table 2:** Genetic variants of *EGLN1*/PHD2 gene and the risk of lung cancer

	Patients	Controls	OR*	95%CI	p-Value	OR**	95%CI	p-Value	OR***	95%CI	p-Value
D4E											
Wildtype	174	192	1.00		0.0012	1.00		0.0109			0.0094
Heterozygous	35	20	**2.12**	**(1.16-3.88)**		**2.06**	**(1.13-3.7)**		**1.91**	**(1.06-3.5)**	
Homozygous	5	1	8.35	(0.82-84.98)		6.74	(0.76-60)		4.97	(0.6-43.5)	
Hetero/Homo	40	21	**2.35**	**(1.31-4.22)**		**2.26**	**(1.27-4.0)**		**2.06**	**(1.16-3.6)**	
C127S											
Wildtype	74	101	1.00		0.0002	1.00		0.0018			
Heterozygous	102	94	**1.60**	**(1.05-2.42)**		**1.52**	**(1.00-2.3)**		1.43	(0.94-2.2)	0.0022
Homozygous	38	18	**3.03**	**(1.59-5.78)**		**3.09**	**(1.62-5.9)**		**2.75**	**(1.44-5.2)**	
Hetero/homo	140	112	**1.83**	**(1.24-2.72)**		**1.76**	**(1.19-2.6)**		**1.64**	**(1.1-2.43)**	

* adjusting for ethnicity (Tibetan/non-Tibetan)

** adjusting for religion (Hindu/not Hindu)

*** adjusting for rs142764723 and rs117813469

**Table 3:** *EGLN1*/PHD2 D4E rs186996510 genotype and the risk of lung cancer in Nepal

	Patients		Controls		OR*	OR**	OR***
	Wildtype	Carrier	Wildtype	Carrier	OR(95%CI)	OR(95%CI)	OR(95%CI)
**Hetero/Homozygote**	174	40	192	21	**2.35 (1.31-4.22)**	**2.26 (1.27-4.02)**	**2.04 (1.13-3.71)**
**By sex**							
Men	94	20	121	8	**2.35 (1.31-4.22)**	**3.49 (1.45-8.42)**	**3.34 (1.36-8.23)**
Women	80	20	71	13	**4.98 (1.8-13.74)**	1.46 (0.67-3.17)	1.11 (0.48-2.55)
**By smoking status**							
Never smoker	18	4	71	7	**2.25 (1.13-4.48)**	2.24 (0.58-8.67)	
Ever smoker	156	36	121	14	1.33 (0.61-2.89)	**2.15 (1.1-4.22)**	
**By age**							
Less than 60 years	66	12	116	9	2.56 (0.65-10.03)	2.43 (0.96-6.14)	2.46 (0.94-6.39)
60+ years	108	28	76	12	2.48 (0.98-6.28)	1.81 (0.85-3.86)	1.6 (0.74-3.44)
**By residential area**							
Plain	97	14	139	13	**3.31 (1.19-9.19)**	1.58 (0.71-3.52)	1.46 (0.63-3.37)
Hills, mountains	77	26	53	8	1.92 (0.88-4.2)	**2.72 (1.09-6.82)**	2.31 (0.94-5.67)
**By resident**							
West	102	32	87	7	**4.5 (1.8-11.26)**	**4.19 (1.73-10.15)**	**4.24 (1.69-10.63)**
East	72	8	105	14	1.58 (0.71-3.51)	0.87 (0.35-2.21)	0.75 (0.29-1.93)
**By religion**							
Hindu	162	36	174	14	**2.77 (1.44-5.32)**		**2.68 (1.35-5.34)**
Not-Hindu	12	4	18	7	1.44 (0.27-7.83)		0.83 (0.2-3.56)

* adjusting for Tibetan ethnicity

** adjusting for Hindu religion

*** adjusting for smoking status

We also explored whether the association differed depending on the cancer type and disease stage. These analyses were limited due to smaller numbers of cases. For lung squamous cell carcinoma, the odds ratio (ORs) for the *EGLN1*^D4E^ carriers were 3.79 (95%CI=1.79, 8.01) adjusted for Tibetan ethnicity, 3.8 (95%CI-1.78, 8.11) when adjusted for Hindu religion and 3.38 (95%I=1.59, 7.21) when adjusted for smoking status. The ORs for the other histological subtypes (small cell, adenocarcinoma) were not significant. Among patients with stage 1 and 2 lung cancer, the ORs for the *EGLN1*^D4E^ carriers were 3.22 (95%CI=1.47, 7.04) when adjusted for Tibetan ethnicity, 3.24 (95%CI=1.47, 7.12) when adjusted for Hindu religion and 2.56 (95%CI=1.17, 5.58) when adjusted for smoking status. Among those with stage 3 and 4 disease, the ORs for the *EGLN1*^D4E^ carriers were 2.43 (95%CI=1.07, 5.53) when adjusted for Tibetan ethnicity, 2.39 (95%CI=1.05, 5.41) when adjusted for Hindu religion and 2.01 (95%CI=0.88, 4.59) when adjusted for smoking status.

To select candidate functional SNPs in the *EPAS1* region, we performed the Composite of Multiple Signals (CMS) test [19]. The CMS test combined five independent tests (XP-EHH, iHS, diHH, FST, and DAF) to improve the power to detect recent positive selection. Using simulations, the log-posterior probability that the observed data under the positive selection were calculated for each test; then these log-posterior probabilities were summed together to generate the final CMS score. Our CMS test used the whole genome sequences of 17 Tibetans [5]. The details of the CMS test are described elsewhere [5].

The *EPAS1* haplotype originated from an archaic Denisovan-like population; however, this selected haplotype contains both Denisovan-like and non-Denisovan-like variants, suggesting a slow decay of the ancestral haplotype. We selected ten SNPs with the highest CMS scores spanning 200 kb of *EPAS1* region for genotyping (rs113305133, rs149306391, rs188801636, rs61151542, rs77111769, rs373417600, rs150877473, rs142764723, rs117813469, rs13005507) according to the following selection criteria: 1) no pair of SNPs had an R^2^ value greater than 0.9; 2) five SNPs (rs113305133, rs188801636, rs61151542, rs77111769 and rs149306391) were present in the Denisovan reference genome and five SNPs (rs117813469, rs13005507, rs150877473, rs142764723 and rs373417600) were absent from the Denisovan reference genome.

As shown in Table 4 and 5, we observed an odds ratio of greater than two for two of the ten *EPAS1* variants, rs117813469 (p=0.050) and rs142764723 (p=0.025). However, the associations were not significant after correcting for multiple comparisons (p=0.12).

**Table 4:** Genetic variants of *EPAS1*/HIF-2α gene and the risk of lung cancer

	Patients	Controls	OR*	95%CI	OR**	95%CI
**rs117813469**						
GG	186	198				
GC/CC	28	15	**2.25**	**(1.14-4.46)**	**2.12**	**(1.09-4.14)**
**rs130005507**						
CC	160	158				
CG/GG	54	55	0.98	(0.63-1.53)	0.97	(0.63-1.5)
**rs11305133**						
AA	191	197				
AG/GG	23	16	1.67	(0.84-3.34)	1.54	(0.79-3.02)
**rs150877473**						
CC	188	192				
CG/GG	26	21	1.49	(0.79-2.81)	1.34	(0.72-2.49)
**rs188801636**						
AA	190	191				
AG/GG	23	19	1.40	(0.72-2.72)	1.28	(0.67-2.45)
**rs77111769**						
TT	184	194				
TC/CC	30	19	1.82	(0.98-3.41)	1.74	(0.94-3.23)
**rs142764723**						
TT	189	189				
TC/CC	25	24	**2.53**	**(1.33-4.82)**	**2.32**	**(1.24-4.34)**
**rs149306391**						
GG	188	193				
GC/CC	26	20	1.58	(0.83-3.02)	1.44	(0.77-2.71)
**rs373417600**						
GG	199	202				
GC/CC	15	11	1.58	(0.69-3.64)	1.47	(0.65-3.29)

* adjusting for ethnicity (Tibetan/non-Tibetan)

** adjusting for religion (Hindu/not Hindu)

**Table 5:** *EPAS1*/HIF-2α test (EMP1 is the point-wise p-value, EMP2 is the family-wise p-value (corrected)

EPAS1 SNPs	EMP1	EMP2
rs373417600	0.5415	0.9451
rs113305133	0.1928	0.5095
rs149306391	0.1848	0.5155
rs188801636	0.4066	0.8811
rs150877473	0.2957	0.7812
rs61151542	0.4186	0.8871
rs77111769	0.0345	0.1618
rs13005507	0.4565	0.9550
rs117813469	0.0500	0.1908
rs142764723	0.0250	0.1179

## DISCUSSION

HIF pathway plays a crucial role in Warburg effect, a unique metabolic feature of cancer cell metabolism. In our case-control study, we evaluated association between lung cancer and Tibetan-specific evolutionary selected *EGLN1* variants, *EGLN1*^D4E^ and *EGLN1*^C127S^ and ten *EPAS1* SNPs with strong evidence of positive selection. We found that the adaptive Tibetan *EGLN1* haplotype was associated with a two-fold increase in the risk of lung cancer (p=0.0012 for D4E, p=0.0002 for C127S, Table 2). In addition, we observed a two-fold increase in the risk of lung cancer for two of ten interrogated *EPAS1* SNPs, although the associations were not significant after correction for multiple testing.

Lung cancer is the most common cancer worldwide and generally can be divided into two categories: non-small-cell lung cancer (adenocarcinoma, squamous cell cancer and large cell carcinoma) and small cell lung cancer. The morphology and molecular profiles are distinct for each subtype but the recent discoveries of mutations in candidate driver genes, e.g. epidermal growth factor receptor (*EGFR*), anaplastic lymphoma kinase (*ALK*), or c-ROS oncogene1 (*ROS1*), brought a deeper understanding of the lung cancer genome [20]. Nevertheless, the role of hypoxia in the transformation of lung epithelial cells and its role in the tumor supporting microenvironment is not completely elucidated. Surprisingly, the overexpression of the HIF hydroxylases PHD1, PHD2, PHD3 individually and collectively were found as indicators of poor prognosis in non-small cell lung cancer, with PHD2 as the most significant prognostic marker [21]. The silencing of PHD2 in cancer cells provided conflicting data, since when increased [22] or decreased [23], tumor growth was observed and was attributed to different molecular mechanisms. The PHD2 haplodeficiency reduced metastasis without affecting tumor growth by decreased activation of cancer-associated fibroblasts [24] indicating the number of HIF-independent roles of PHDs.

Our data cannot address the specific role of *EGLN1* and *EPAS1* genetic variants in lung cancer initiation, progression, or survival. We cannot rule out the possibility that the inheritance of the *EGLN1*^D4E/C127S^ variants may in fact be associated with longer survival and more indolent clinical course and thus a higher likelihood of detection while those without the *EGLN1*^D4E/C127S^ variant, perhaps living in the more remote isolate area of Nepal, may never reach the referral center to undergo diagnostic tests because of their impending rapid clinical course and/or feeble clinical status precluding the travel. Alternatively, the differential rate of acquiring or eliminating *EGFR*, *ALK*, or *ROS1* lung cancer somatic mutations may be also influenced by the inheritance of the *EGLN1*^D4E/C127S^ variant which modulates HIFs-stability. In addition, in Asian patients with adenocarcinoma histology, an *EGFR* mutation is found to be present at twice the rate of other populations, reflecting the underlying genetic differences [25]. Only a prospective study taking into account these clinical and molecular markers can answer these lung cancer covariant questions.

However, we conclude that high-altitude adapted Tibetan variants in the *EGLN1* and possibly *EPAS1* regions are associated with the modified lung cancer risk, and that the role of these Tibetan evolutionary selected haplotypes needs to be further elucidated. The evolutionary-selected HIF signaling pathways in Tibetans provide a unique opportunity to gain a deeper understanding of cancer pathophysiology in this minority population subjected to unique evolutionary pressures.

## MATERIALS AND METHODS

### Sample acquisition

A hospital-based case-control study was conducted at the B. P. Koirala Memorial Cancer Hospital (BPKMCH) in the city of Bharatpur, in the Chitwan district (Nepal). In this study, we included 214 lung cancer cases (ICD-O2 codes C33 and C34), and 213 controls matched on age (±five years), sex and geographical area of residency (mountain, hilly and plain region). All eligible cases were recruited as soon as possible after initial diagnosis of lung cancer, with a target interval of one day between initial diagnosis and recruitment, and a maximum interval of three months to ensure that very few cases are not recruited because they die before there is an opportunity to interview them. The source of controls were visitors of non-lung cancer patients at the hospital. A standardized questionnaire was administered to all study participants by trained staff members, who collected data on demographic and socioeconomic status, family history of cancer, tobacco and alcohol consumption habits, dietary factors, occupation, residential history and usage of different chewing products available locally. All study subjects gave informed consent. This study has been approved by the IRB committees at the University of Utah, University of Maryland and the Nepal Health Research Council.

### DNA isolation

Genomic DNA was isolated from whole blood using Blood & Cell Culture DNA Mini Kit (Qiagen, Germantown, MD) according to manufacturer's protocol, standardized to 100 ng/μL.

### *EGLN1/*PHD2 genotyping - *EGLN1*^D4E^ high resolution melting genotyping

Because the *EGLN*1 haplotype is located in a GC-rich region and is difficult to detect by next-generation or dideoxynucleotide sequencing, we developed a high resolution melting assay suitable for analyzing limited amounts of DNA [9]. Double layer capillary method was used to perform polymerase chain reaction and high resolution melting of *EGLN*1/PHD2, D4E (rs186996510) using LightScanner 32 capillary real-time instrument (BioFire Defense) in 5 μL reactions separated by 2 μL of liquid wax (Chill-out 14, MJ Research). The 5 μL reaction was prepared using 0.5 μM forward primer (ACGCTCTCACGCCGCCATGGCCAATGA), 0.5 μM reverse primer (GCCGGGCCCGCCGCT), 0.2 U Klen*Taq* DNA Polymerase (JemBiotech), 44 ng antibody clone 8C1 (E-enzyme), 50 mM Tris pH 8.3, BSA 2.5 ug, 2 mM MgCl_2_, 0.2 mM dNTP, 1x LCGreen plus (BioFire Defense), 1 M Betaine (Sigma-Aldrich) and 20 ng of template DNA. The following PCR conditions were used: 98°C for 30 seconds, and 50 cycles of 2 seconds at 98°C, 4 seconds at 70°C and 4 seconds annealing/extension at 75°C. After PCR, high resolution melting was performed from 65°C to 95°C with continuous acquisition at a ramp rate of 0.3°C/sec.

### *EGLN1/*PHD2 genotyping - *EGLN1* exon one Sanger-based genotyping

We subsequently improved the Sanger sequencing method for *EGLN1* genotyping. This improved Sanger method now allows the analysis of this genetic region without requiring the LightScanner 32 capillary real-time instrument (BioFire Defense). All samples were examined by both methods with complete concordance of the results. The 5’ portion of *EGLN1* exon one (c.118 – 471) was amplified using Platinum® *Taq* DNA Polymerase (Life Technologies), FailSafe™ PCR 2X PreMix “J” (Epicentre, Madison, WI, USA) and M13-tailed primers: 5’-TGT AAA ACG ACG GCC AGC ATG GCG CAG TAA CG and 5’-CAG GAA ACA GCT ATG ACC TGG AAC AGC GAT GAG. The PCR conditions were: 98°C for 30 secs, and ten cycles of 20 seconds at 98°C, 20 seconds annealing at 62°C minus 0.5°C/cycle and 1-minute extension at 72°C, followed by 25 cycles of 20 seconds at 98°C, 20 seconds annealing at 57°C and 1-minute extension at 72°C. Amplification products were purified using EXOSapIt (Affymetrix, Santa Clara, CA, USA) to remove excess PCR primers and dNTPs. Sequencing was performed using M13 primers and BigDye Terminator v3.1 Cycle Sequencing Kit (Life Technologies, Grand Island, NY, USA) following manufacturer's published protocol using a 3130xl Genetic Analyzer (Life Technologies). Sequence data was analyzed using Mutation Surveyor software (Softgenetics, State College, PA, USA).

### *EPAS1*/HIF-2α SNPs genotyping

The ten SNPs were amplified using multiplex touch-down PCR and analyzed by single nucleotide extension (SNE) protocol (Life Technologies). Briefly, multiplex PCR was run with Platinum® *Taq* DNA Polymerase (Life Technologies), FailSafe™ PCR 2X PreMix “D” (Epicentre) and the primers listed in Supplementary Table 1. The PCR conditions were: 98°C for 30 sec, and ten cycles of 20 seconds at 98°C, 20 seconds annealing at 62°C minus 0.5°C/cycle and 45 seconds extension at 72°C, followed by 25 cycles of 20 seconds at 98°C, 20 seconds annealing at 57°C and 45 seconds extension at 72°C. Amplification products were purified using EXOSapIt (Affymetrix) to remove excess PCR primers and dNTPs. Amplified products were then added to SNaPshot Multiplex System (Life Technologies) reagents according to manufacturer's protocol on a GeneAMp PCR system 9700 (Applied Biosystems, Foster City, CA). Following SNE cycling reaction excess ddNTPs were removed using Shrimp Alkaline Phosphatase (Affymetrix, Santa Clara, CA, USA) and analyzed using a 3130xl Genetic Analyzer (Applied Biosystems). Primers for PCR were designed using MPprimer [26]. Primers for SNE were manually selected and screened using Primer Designer (v.5) software (Scientific & Educational Software, Morrisville, NC, USA). The SNE primers were designed to lengths between 15 and 60 nucleotides. Fragment analysis was conducted using GeneMarker software (Sofgenetics).

### Statistical analysis

Unconditional logistic regression was used to estimate the odds ratios (OR) and their 95% confidence intervals (95%CI), to evaluate the risk of lung cancer according to various genetic variants. Given the extensive linkage disequilibrium in the *EPAS1* region among Tibetans, a Bonferroni correction would be overly conservative. Therefore, we performed a randomization test to estimate the family wise error rate for the ten SNPs in the *EPAS1* region. Specifically, we computed the max(T) permutation p-value for Cochran–Armitage test for trend using PLINK v1.07.

## SUPPLEMENTARY MATERIALS TABLE



## References

[1] Beall C, Brittenham G, Strohl K, Blangero J, Williams-Blangero S, Goldstein M, Decker M, Vargas E, Villena M, Soria R, Alarcon A, Gonzales C (1998). Hemoglobin concentration of high-altitude Tibetans and Bolivian Aymara. Am J Phys Anthropol.

[2] Moore L (2001). Human genetic adaptation to high altitude. High Alt Med Biol.

[3] Bigham A, Mao X, Brutsaert T, Wilson M, Julian C, Parra E, Akey J, Moore L, Shriver M (2009). Identifying positive selection candidate loci for high-altitude adaptation in Andean populations. Hum Genomics.

[4] Simonson T, McClain D, Jorde L, Prchal J (2011). Genetic determinants of Tibetan high-altitude adaptation. Hum Genet.

[5] Hu H, Petousi N, Glusman G, Yu Y, Bohlender R, Downie J, Roach J, Cole A, Lorenzo F, McClain D, Rogers A, Brunkow M, Cavalleri G, Hood L, Prchal J, Jorde L (2016). Evolutionary history of Tibetans inferred from whole-genome sequencing. PLOS Genetics.

[6] Yi X, Liang Y, Huerta-Sanchez E, Jin X, Cuo Z, Pool J, Xu X (2010). Sequencing of 50 human exomes reveals adaptation to high altitude. Science.

[7] Simonson T, Yang Y, Huff C, Yun H, Qin G, Witherspoon D, Bai Z, Lorenzo F, Xing J, Jorde L, Prchal J, Ge R (2010). Genetic Evidence for High-Altitude Adaptation in Tibet. Science.

[8] Peng Y, Yang Z, Zhang H, Cui C, Qi X, Luo X, Tao X, Wu T, Basang Ouzhuluobu, Danzengduojie Ciwangsangbu, Chen H, Shi H, Su B (2011). Genetic variations in Tibetan populations and high-altitude adaptation at the Himalayas. Mol Biol Evol.

[9] Lorenzo F, Huff C, Myllymäki M, Olenchock B, Swierczek S, Tashi T, Gordeuk V, Wuren T, Ri-Li G, McClain D, Khan T, Koul P, Guchhait P, Salama M, Xing J, Semenza G (2014). A genetic mechanism for Tibetan high-altitude adaptation. Nat Genet.

[10] Song D, Li L, Arsenault P, Tan Q, Bigham A, Heaton-Johnson K, Master S, Lee F (2014). Defective Tibetan PHD2 binding to p23 links high altitude adaption to altered oxygen sensing. J Biol Chem.

[11] Beall C, Cavalleri G, Deng L, Elston R, Gao Y, Knight J, Li C, Li J (2010). Natural selection on EPAS1 (HIF2alpha) associated with low hemoglobin concentration in Tibetan highlanders. Proc Natl Acad Sci U S A.

[12] Huerta-Sánchez E, Jin X, Z Asan Bianba, Peter BM, Vinckenbosch N, Liang Y, Yi X, He M, Somel M, Ni P, Wang B, Ou X (2014). Altitude adaptation in Tibetans caused by introgression of Denisovan-like DNA. Nature.

[13] Jeong C, Alkorta-Aranburu G, Basnyat B, Neupane M, Witonsky D, Pritchard J, Beall C, Di Rienzo A (2014). Admixture facilitates genetic adaptations to high altitude in Tibet. Nature Communications.

[14] Manalo D, Rowan A, Lavoie T, Natarajan L, Kelly B, Ye S, Garcia J, Semenza G (2005). Transcriptional regulation of vascular endothelial cell responses to hypoxia by HIF-1. Blood.

[15] Semenza G (2012). Hypoxia-Inducible Factors in Physiology and Medicine. Cell.

[16] Shimoda L, Semenza G (2011). HIF and the lung: role of hypoxia-inducible factors in pulmonary development and disease. Am J Respir Crit Care Med.

[17] Shen C, Beroukhim R, Schumacher S, Zhou J, Chang M, Signoretti S, Kaelin WJ (2011). Genetic and functional studies implicate HIF1α as a 14q kidney cancer suppressor gene. Cancer Discov.

[18] Zhang H, Lu H, Xiang L, Bullen J, Zhang C, Samanta D, Gilkes D, He J, Semenza G (2015). HIF-1 regulates CD47 expression in breast cancer cells to promote evasion of phagocytosis and maintenance of cancer stem cells. Proc Natl Acad Sci U S A.

[19] Grossman SR, Shlyakhter I, Karlsson EK, Byrne EH, Morales S, Frieden G, Hostetter E, Angelino E, Garber M, Zuk O, Lander ES, Schaffner SF, Sabeti PC (2010). A composite of multiple signals distinguishes causal variants in regions of positive selection. Science.

[20] Sholl L (2015). Biomarkers in lung adenocarcinoma: a decade of progress. Arch Pathol Lab Med.

[21] Andersen S, Donnem T, Stenvold H, Al-Saad S, Al-Shibli K, Busund L, Bremnes R (2011). Overexpression of the HIF Hydroxylases PHD1, PHD2, PHD3 and FIH Are Individually and Collectively Unfavorable Prognosticators for NSCLC Survival. PLoS One.

[22] Bordoli M, Stiehl D, Borsig L, Kristiansen G, Hausladen S, Schraml P, Wenger R, Camenisch G (2011). Prolyl-4-hydroxylase PHD2- and hypoxia-inducible factor 2-dependent regulation of amphiregulin contributes to breast tumorigenesis. Oncogene.

[23] Klotzsche-von Ameln A, Muschter A, Mamlouk S, Kalucka J, Prade I, Franke K, Rezaei M, Poitz DM, Breier G, Wielockx B (2011). Inhibition of HIF prolyl hydroxylase-2 blocks tumor growth in mice through the antiproliferative activity of TGFβ. Cancer Res.

[24] Kuchnio A, Moens S, Bruning U, Kuchnio K, Cruys B, Thienpont B, Broux M, Ungureanu A, R Leite de Oliveira, Bruyère F, Cuervo H, Manderveld A, Carton A, Hernandez-Fernaud J, Zanivan S, Bartic C (2015). The Cancer Cell Oxygen Sensor PHD2 Promotes Metastasis via Activation of Cancer-Associated Fibroblasts. Cell Reports.

[25] Shi Y, Au J, Thongprasert S, Srinivasan S, Tsai C, Khoa M, Heeroma K, Itoh Y, Cornelio G, Yang P (2014). A prospective, molecular epidemiology study of EGFR mutations in Asian patients with advanced non-small-cell lung cancer of adenocarcinoma histology (PIONEER). J Thorac Oncol.

[26] Zhiyong S, Wubin Q, Wen W, Yiming L, Yonghong W, Zhifeng L, Xingyi H, Xiaolei W, Dongsheng Z, Chenggang Z (2010). MPprimer: a program for reliable multiplex PCR primer design. BMC Bioinformatics.

