# Supplementary material for: Evolutionary selected Tibetan variants of HIF pathway and risk of lung cancer
Source: Oncotarget. 2016 Dec 28;8(7):11739–47. doi: 10.18632/oncotarget.14340 (PMC5355300; doi:10.18632/oncotarget.14340)
Supplement: Supplementary file 1 [file oncotarget-08-11739-s001.pdf]

Evolutionary selected Tibetan variants of HIF pathway and risk of lung cancer

SUPPLEMENTARY TABLE

Supplementary Table S1: Amplification and SNE primers *EPAS1*/HIF-2α region of Chromosome 2

Target	Forward Primer	Reverse primer	SNE primer
rs113305133	TATGCAAGGTTAGTGAAAGCCC	CATGCTATTCCAAGGTTTCCCC	T(7)GCCCCTT ACCTTCACCCC
rs149306391	GAGTATTCGCCTAGGAAGAGCA	TCACTGTTCCCCTTTCACAGAT	AGATTGTTATTGT CATTGTTCTTCT TTCACAGATCTG GAAACAGACACTT
rs188801636	AAAGGCCTTATCCTCTAGCCTG	ACATCATTTCTCCAGAAGCCTA	TACATCATTTCTCCA GAAGCCTATA TTTAATATAA
rs61151542	TCCCGTTAATTACAGAGCTGGT	AACTCAGATTTCAGTGCCTTCG	T(14)CCTTGTGTTT TCCTCAAATACTACAT
rs77111769	TTTGACCTTCCCCATTTCCAAC	CAGTTCTGGGCTGTATTATGCC	T(23)CCCTTCTTAG GCAAACTAATGG
rs373417600	AGGATGAAAACGCTCATCCTTG	ACTCTGTCTCTCCTGTTAAGCC	ATAAGGTTTAGT TTGTTCCCCACTTACG TAACTGCTTTTAATAAT GTGCCTTGAAAACTT
rs150877473	GAGTCCCAGGTGTAGGGTAAC	GGGCAGTTGTTGTAGACTTTCA	CGGCTCCATGTCT GACCCTT CCACGCCTGT
rs142764723	AGAAAGCTTGTACAGGGGAACT	GTTTTTCCTGTGCTGTTCTTGC	GAATCATGGG CTTGGGTTTTTCCTG TTTTGTTCTTGCGC TAGTGAATAAGTCTCA
rs117813469	AACCTGGACTCAGGAAGATCAG	CACATCTCTTCCTTCCATCGC	TGTCCTGGCCCTCGCT
rs13005507	GTTGGGCCCAGAGTTGATCT	AGATGGAGGGGATGCTTTGATT	TGTTGATCTG CTGACCCACTT
